# A Comparison Study of Classifier Algorithms for Cross-Person Physical Activity Recognition

**DOI:** 10.3390/s17010066

**Published:** 2016-12-30

**Authors:** Yago Saez, Alejandro Baldominos, Pedro Isasi

**Affiliations:** Department of Computer Science, Universidad Carlos III de Madrid, 28911 Leganés, Spain; abaldomi@inf.uc3m.es (A.B.); isasi@ia.uc3m.es (P.I.)

**Keywords:** physical activity recognition, classification, machine learning, deep learning, biomedical signal processing, time series analysis

## Abstract

Physical activity is widely known to be one of the key elements of a healthy life. The many benefits of physical activity described in the medical literature include weight loss and reductions in the risk factors for chronic diseases. With the recent advances in wearable devices, such as smartwatches or physical activity wristbands, motion tracking sensors are becoming pervasive, which has led to an impressive growth in the amount of physical activity data available and an increasing interest in recognizing which specific activity a user is performing. Moreover, big data and machine learning are now cross-fertilizing each other in an approach called “deep learning”, which consists of massive artificial neural networks able to detect complicated patterns from enormous amounts of input data to learn classification models. This work compares various state-of-the-art classification techniques for automatic cross-person activity recognition under different scenarios that vary widely in how much information is available for analysis. We have incorporated deep learning by using Google’s TensorFlow framework. The data used in this study were acquired from PAMAP2 (Physical Activity Monitoring in the Ageing Population), a publicly available dataset containing physical activity data. To perform cross-person prediction, we used the leave-one-subject-out (LOSO) cross-validation technique. When working with large training sets, the best classifiers obtain very high average accuracies (e.g., 96% using extra randomized trees). However, when the data volume is drastically reduced (where available data are only 0.001% of the continuous data), deep neural networks performed the best, achieving 60% in overall prediction accuracy. We found that even when working with only approximately 22.67% of the full dataset, we can statistically obtain the same results as when working with the full dataset. This finding enables the design of more energy-efficient devices and facilitates cold starts and big data processing of physical activity records.

## 1. Introduction

Physical activity recognition has numerous applications in fields, such as medical monitoring, healthy aging, active living and rehabilitation, and so on. For many years, the medical research literature has extensively explored the positive effects of physical activity on health. These positive effects accrue to humans regardless of age. For instance, in the early stages of physical human development, Janssen and Leblanc [1] explored the benefits of physical activity in school-aged children and youth; Merglen et al. [2] showed the advantages of weekly sports practice for adolescent wellbeing; and Biddle and Asare [3] studied the association between mental illnesses and lack of physical activity. Regarding the latter stages of human development, the work by Chodzko-Zajko [4] focused on the benefits of physical activity in healthy aging and concluded that physical activity reduces the risk factors of chronic diseases.

Activity recognition is a field that has gained increasing interest given the availability of commodity hardware devices (i.e., smartphones, wearable devices, etc.). These devices contain a set of sensors, such as accelerometers, gyroscopes, and GPS, that can provide the basic data required to perform activity recognition, as described by [5], who provided a complete overview of the past, present and future of pervasive sensing technologies and their applications for health monitoring.

Proof of the increasing interest in activity monitoring can be found in the large number of inventions and patents in recent years that aim at monitoring and quantifying people’s physical activity [6,7] and (more recently) at recognizing the activity being performed [8]. In some cases, these provide customized personal training features [9,10]. In addition, healthcare-related cell phone apps have also become popular in the last few years. Some of the larger cell phone manufacturers have included specific sensor hardware in their cell phones (e.g., the Bosch (Stuttgart, Germany) BMA280 accelerometer or InvenSense (San Jose, CA, USA)’s six-axis MPU-6700).

Physical activity recognition is primarily a classification problem [11,12]. Physical activity recognition systems are not new; there are previous related works that have been developed since more than two decades [13]. However, the arrival of new technologies to wearable sensors and mobile devices has enabled novel applications in different areas, such as healthcare, sport or security, among others, and at this moment, there are many open problems to be tackled. Recent surveys [14,15,16] have reviewed in detail the most relevant previous works conducted in this field.

According to [14], research in human activity recognition falls into seven different categories: (1) selection of attributes and sensors; (2) obtrusiveness; (3) data collection protocol; (4) recognition performance; (5) energy consumption; (6) processing; and (7) flexibility. This article falls mainly into the last category: flexibility to support new users without the need for collecting additional data of the user and re-training the system. This topic, also known as cross-person activity recognition, has been previously addressed by many other works, such as [17,18] and, more recently, [19].

The contributions we want to make with respect to previous works are: (1) to conduct a complete comparison of the most relevant out-of-the-box scikit-learn classifiers in this field and how they compare with other techniques from previous works; (2) to include a deep learning approach into the comparison and analyze how it compares with common machine learning classifiers; and (3) to include an additional study of how these algorithms perform with limited data, which implies less energy consumption needed and addresses the fifth issue described in [14].

### Motivations

**Figure 1:**
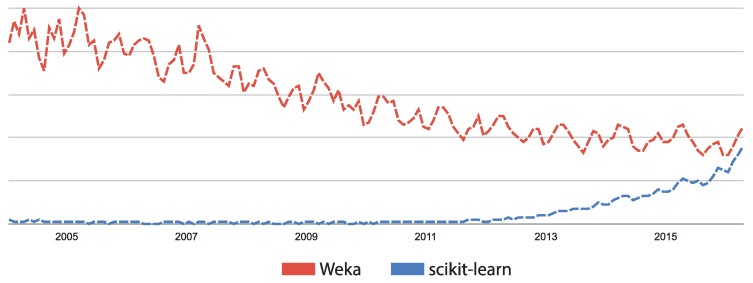
Search interest through time of both WEKA and scikit-learn. Source: Google Trends [37].

As far as we know, this is the first work using the scikit-learn library for physical activity recognition. Most of the existing research works are based on WEKA [11,20,21,22], MATLAB [23], or proprietary developments, such as the work presented by [24], among many others. The motivation for using the scikit-learn library in this paper is two-fold. First, this library was implemented by experts; all algorithms are peer reviewed; and it is widely used and has been gaining in popularity in the last few years (see Figure 1). Second, using this library in conjunction with a public dataset (such as PAMAP2 (Physical Activity Monitoring in the Ageing Population) [25]) ensures that all of the experiments conducted in this work can be easily replicated and can be used as a benchmark.

Nowadays, one of the trending research lines is applying deep learning techniques to extract features and classify in one step, starting from the raw dataset. This approach has been addressed in a few works; a representative set can be found in [26,27,28,29,30,31]. There is an interesting comparison of different deep learning techniques applied to the PAMAP dataset (among others) in [32]. Results found in that work with deep learning techniques are outperformed by the ones achieved in this work with machine learning classifiers, which suggests that more research and comparison still need to be done. Since we have the comparison of [32], the motivation for including deep learning techniques into our comparison is to analyze how these techniques compare in performance with common state-of-the-art machine learning classification techniques, but when working with the classic approach of signals already transformed into the frequency domain.

Last, but not least, one important research topic when working with sensors embedded in devices running on batteries is how to optimize their energy consumption [33,34,35,36]. In physical activity recognition with wearable devices, there is an important tradeoff between accuracy and power consumption. Just collecting and sending all information gathered from all available sensors through the data network have a great impact on energy consumption. However, if we are able to achieve acceptable classification accuracies (higher than 90%) using much less information coming from sensors, then we could use only a sample of what is available in order to save energy and time. In this work, we have tested with nine different sample sizes, starting from 10% to 0.001%.

## 2. Methodology

In this paper, we compare classic, state-of-the-art machine learning techniques and deep learning techniques to achieve cross-person prediction of the physical activity performed by a subject. When performing cross-person predictions, the distribution of data is heavily affected by the various users; consequently, the prediction performance degrades when a model trained on one person is used on others.

Most works in the field of human activity recognition follow a so-called activity recognition chain (ARC). This sequence of steps was extensively described by [38] as a general-purpose framework for acquiring data samples and building and evaluating activity recognition systems.

**Figure 2:**
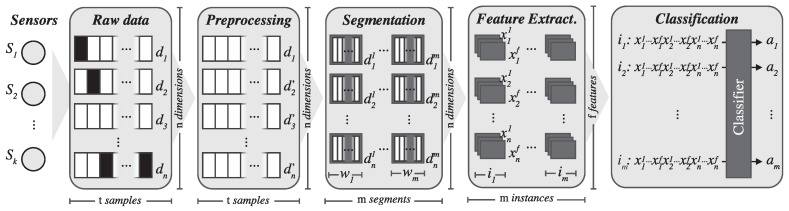
Steps involved in the activity recognition chain (ARC) that range from data collection to classification.

Figure 2 shows the different steps involved in the ARC. These include data acquisition from sensors, signal preprocessing, signal segmentation, feature extraction and training a classification model. In this section, we will thoroughly describe each of these steps.

### 2.1. Data Acquisition

The first stage of the ARC involves data acquisition: the design and development of a process to gather data about the subjects performing physical activities. While these data can be of various types, a frequent approach consists of placing different sensors on subjects’ bodies to obtain data about their motions. These devices range from smartphones and wearable devices, such as activity wristbands, to specific sensors attached directly to the body.

Besides the devices themselves, the data acquisition stage must also define a protocol that must be strictly followed to guarantee the validity of the obtained samples and consistency between different subjects.

In this paper, we use the physical activity monitoring dataset PAMAP2, introduced by [24,25,39,40,41,42,43] and publicly available at the University of California, Irvine (UCI) Machine Learning Repository. Selecting this dataset enables future replications of the experiments in this paper by the scientific community.

The PAMAP2 dataset contains labeled information about the physical activity performed by 9 different subjects wearing heart rate monitors and three Colibri wireless inertial measurement units (IMUs). This section provides further detail about the subjects’ profiles, the wearable devices and the protocol used to acquire the physical activity data.

#### 2.1.1. Subjects

The PAMAP2 dataset provides physical activity data about 9 different subjects. The subjects who participated in the data acquisition protocol are mainly employees or students at DFKI (German Research Centre for Artificial Intelligence) and consist of 1 female and 8 males, aged from 27.22 ± 3.31 years. Their average body mass index (BMI) ranges from 25.11 ± 2.62 kg·m${}^{-2}$. Further details about each subject are provided in Table 1.

In this work, we omitted Subject 9, as we observed that, due to communication problems, most of the data for that subject were either recorded incorrectly or lost.

#### 2.1.2. Devices

For the data acquisition stage, three Colibri^®^ wireless inertial measurement units (IMUs) were used. These devices are fastened to the wrist on the dominant arm, to the chest and to the ankle on the dominant side, respectively. The IMUs are commercially available through Trivisio Prototyping GmbH (Trier, Germany), an engineering company that specializes in wearable devices. These devices carry 3-axis state-of-the-art sensors that include the following:

- An accelerometer with a scale of ±16 g and a 13-bit resolution
- An accelerometer with a scale of ±6 g and a 13-bit resolution
- A gyroscope with a scale of ${\pm 2000}^{\circ }$/s and a 16-bit resolution
- A magnetometer with a scale of ±1.3 Ga and a 12-bit resolution

Moreover, the devices also feature a temperature sensor with an accuracy of ±0.5 ${}^{\circ }$C. In addition to temperature, the device provides information about its orientation (pitch, roll and yaw). The size of each device is 52 mm × 42 mm × 17 mm; it weighs 41 g and is able to operate in a self-powered manner within a temperature range from 0 to 55 ${}^{\circ }$C and within a 10-m working distance. The IMUs have a sampling frequency of 100 Hz.

The heart rate monitors worn by the subjects were Model BM-CS5SR from BM Innovations GmbH (BM Wireless Ltd.&Co.KG, Hörgertshausen, Germany). These monitors have a frequency of approximately 9 Hz and an estimated data transmission range of approximately 30 m.

The wearable devices were connected to a Viliv S5 UMPC ultra-portable PC (Dynamism Inc., Chicago, IL, USA), configured with an Intel (Santa Clara, CA, USA) Atom Z520 1.33-GHz CPU and 1 GB of RAM. This PC was used both for storing the data obtained from the devices and for labeling the activities performed at every stage of the protocol.

#### 2.1.3. Protocol

All subjects adhered to a protocol describing a sequence of 12 different activities that had to be performed in a specific order, spending a predetermined amount of time on each activity.

The description of the activities to be performed is as follows:

- lying quietly while doing nothing (small movements are allowed).
- sitting in a chair (changing postures is allowed).
- standing still (talking or gesticulating is allowed).
- ironing one or two shirts.
- vacuuming one or two rooms, moving objects if required.
- ascending stairs for a distance of five floors.
- descending stairs for a distance of five floors.
- walking outside with a speed of 4 to 6 km/h.
- Nordic walking on asphaltic terrain using asphalt pads on the walking poles.
- cycling with slow to moderate pace.
- running or jogging outside.
- jumping rope either with both feet at the same time or alternating feet.

Table 2 describes the protocol defined for data collection. The third and fourth columns specify the activity code and the estimated effort by means of the metabolic equivalent (MET), respectively, as provided by Ainsworth et al. [44].

While this protocol should ideally be followed by all subjects, in a real setup, some data are lost or some subjects refuse to perform certain activities, resulting in invalid data in the dataset. Table 3 shows the total time spent by each subject in each activity. It should be noted that the actual collected data differ from the expected collection results according to the protocol. More importantly, this information clearly justifies the exclusion of Subject 9 from this work.

After the data collection stage was complete, data were stored in tabular format using one row for each sample with 54 columns for the attributes:

- 1        timestamp (in s).
- 2        activity.
- 3        heart rate.
- 4 to 20       IMU on wrist.
- 21 to 37  IMU in chest.
- 38 to 54  IMU on ankle.

For each IMU, the data are structured in columns as follows:

- 1        temperature (in ${}^{\circ }$C).
- 2 to 4         3D acceleration data (ms${}^{-2}$), scale ±16 g.
- 5 to 7         3D acceleration data (ms${}^{-2}$), scale ±6 g.
- 8 to 10       3D gyroscope data (rad/s), scale ±2000${\;}^{\circ }$/s.
- 11 to 13  3D magnetometer data (μT), scale ±1.3 Ga.
- 14 to 17  orientation.

### 2.2. Signal Preprocessing

Because the data collection stage was performed in a real-world scenario, the raw data contain noise, invalid data or missing samples. For this reason, preprocessing is an essential phase before performing classification. This section describes the different actions that take place in the preprocessing stage, which receives as input the original raw data and returns a new, transformed dataset.

#### 2.2.1. Remove the Timestamp

The timestamp is not a useful feature for classification, as it is not part of the physiological signals extracted during the data collection stage. Thus, the timestamp should be removed; otherwise, the classifier might learn the protocol sequencing and achieve a high classification accuracy due to this feature.

#### 2.2.2. Remove the Orientation

The authors of the PAMAP2 dataset state that the orientation information it contains is either invalid or not relevant for this data collection effort [18]. For this reason, we also removed the orientation data, consisting of a total of 12 features in the dataset, four per each IMU.

#### 2.2.3. Remove Transitions

After one activity ends and before the next one starts, the dataset contains samples labeled as transition. These samples do not correspond with any activity; instead, they are noise and should be removed.

#### 2.2.4. Estimate Missing Values

In the PAMAP2 dataset, missing values are specified as not-a-number (NaN). For most attributes, the number of missing values is negligible, and these occur due to communication failures between the IMUs and the PC. For heart rate measurements, most missing values are because of the frequency of the heart monitor (9 Hz), compared to the 100 Hz of the IMUs. Therefore, about 8 out of 9 samples have missing values for the subjects’ heart rates.

In all cases, we estimated missing values as the average of the previous and the next available values. We consider this naive approach to be valid because the missing values would not be expected to change significantly within one hundredth of a second (or one ninth of a second in the case of the heart rate).

### 2.3. Signal Segmentation

Initially, the raw data are in the time domain because each sample captures values from the different sensors at a certain point in time. Of course, these tabular data are suitable for training machine learning models, but we expect to achieve higher classification accuracy when the raw data are transformed to capture temporal information as in previous works, such as those by [22,45]. Bearing this consideration in mind, we designed a segmentation stage in which we transform the input data into the frequency domain.

**Figure 3:**
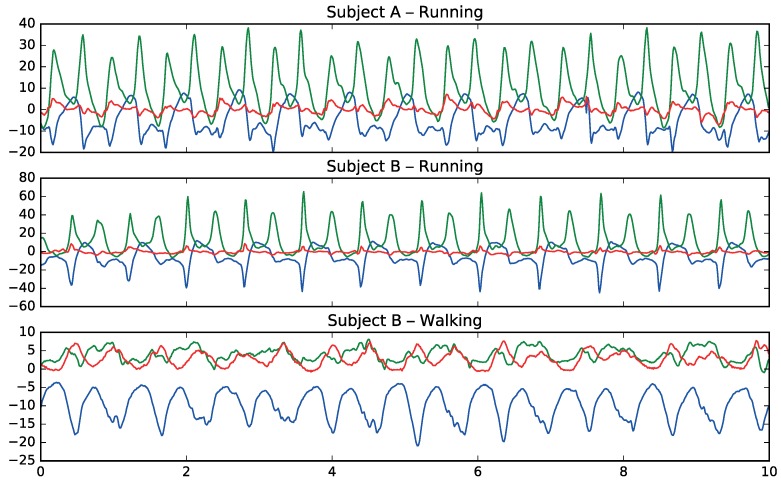
Information from the accelerometer sensor for 2 different activities (running and walking). The unit of the vertical axis is m/s${}^{2}$. The colors are blue (*x*), green (*y*) and red (*z*).

Figure 3 shows how patterns in movement are different between walking and running and how they have common patterns among different subjects for the same activities.

To do this, for each original feature in the dataset, we take a sliding window of a size of 512, corresponding to 5.12 s of physical activity data. We guarantee that these sliding windows contain samples from only one activity. Then, we compute a discrete Fourier transform (DFT) for this signal window using the FFT algorithm. As a result, the signal is transformed into the frequency domain, and the output comprises 512 values.

### 2.4. Feature Extraction

After transforming the input data into the frequency domain by computing the DFT for each sliding window of each attribute, we process the resulting signal to extract features that compose the new transformed dataset.

In Pirttikangas et al. [12], the authors found the most important feature to be the mean acceleration; they did not include mean heart rate or heart rate correlations in their subset of best features. However, other works, such as those by [17,21], concluded that mean heart rate is a significant feature depending on the type of physical activity involved.

In the feature extraction stage, each sliding window generates a new instance. To compose the features of this instance, we compute a statistical summary of the 512 values resulting from applying the DFT; this summary consists of the following values: average, median, variance, maximum, minimum, 25% percentile and the 75% percentile. As a result, each physiological feature in the preprocessed dataset is substituted by these 7 features in the resulting dataset.

In conclusion, we end up with a dataset containing 280 features and one class (the physical activity), where each feature is a value from the statistical summary of the DFT of another feature in the preprocessed dataset.

Regarding feature selection, a wide variety of feature selection methods that can identify which attributes are most important for classifiers can be found in [46]. The selection of significant features is important to reduce the dimensionality of the classification problem and because in several domains (including the medical domain), the identification of discriminative features can contribute significantly to the domain knowledge.

In this study, we decided not to perform feature selection for two reasons. First, the main objective of this work is to evaluate how well state-of-the-art classifiers deal with very small dataset sizes for physical activity recognition. We did not want to reduce the features and the size of the dataset at the same time. Second, as we learned from previous works by [45], feature selection affects different classifiers in different ways; therefore, depending on the feature selection method used, we would either positively or negatively affect some classifiers. To avoid this bias in the results, we would have had to execute many combinations of different feature selection methods with the algorithms and parameters, which would detract from the main goals of this work and affect the generality and understanding of this paper.

### 2.5. Classifier Model Training

The last stage of the activity recognition chain involves the training of a classification model that is able to discriminate the physical activity performed by a certain subject. Our objective is to eventually obtain a classifier that maximizes accuracy (i.e., the hit rate between the predicted and the actual class in a test set).

The resulting classifier is intended to be used in a real-time cross-person prediction system that must be able to accurately predict the activity that a new user is performing. When facing this problem, two different approaches are commonly considered regarding the data feeding the classification model; either (1) the model is subject-independent, and data from a set of different users are used to train the classifier; or (2) the model is subject-group specific, and only historic data from similar users (for example, clustered by age, gender, etc.) are used for training and prediction. While the second approach could potentially provide higher accuracy, it has an important flaw: the system is not able to provide accurate predictions during cold start, when there is not enough information about the users. With cross-person prediction, subject-specific training cannot be performed because we have no data to use for new users. Another interesting and novel approach proposes using template matching to classify sports activities. This approach showed robust classification accuracies when tested on unseen data and when limited training examples were available [47].

For this reason, the classification system we propose features a hybrid approach that is subject-independent during cold start, but eventually turns into a subject-specific model after enough data have been recorded for a particular user. To test this approach, in this paper, we use leave-one-subject-out (LOSO) cross-validation. This evaluation methodology splits the dataset into *N* folds, where *N* is the number of subjects (in this case N=8), using N−1 folds as the training set and the remaining fold as the test set. Through this procedure, we guarantee that the classifier learns nothing about the subject to be predicted, thus decreasing learning bias and achieving accurate results for cross-person prediction. Note that an evaluation methodology that does not use LOSO would provide better results in terms of accuracy [18] because the model would have already encountered instances from the target subject, but its results would not be representative of the actual prediction capabilities during cross-person prediction or during a cold start.

To choose the most adequate classification model, we first completed a preliminary evaluation that compared different supervised learning techniques using increasingly small percentages (e.g., 5%, 1% and 0.1%) of the complete input data. Some techniques provide higher classification accuracies when data are normalized; thus, in some cases, the samples were normalized to the range [0, 1] by applying Equation (1), where ${\hat{v}}^{\left(f\right)}$ is the transformed value, $v^{\left(f\right)}$ is the original value and both values, the maximum and minimum required to calculate the normalization, refer to feature *f*, as shown below:

(1) $${\hat{v}}^{\left(f\right)}=\frac{v^{\left(f\right)}-min^{\left(f\right)}}{max^{\left(f\right)}-min^{\left(f\right)}}$$

To prototype a fast evaluation system, we used Python’s machine learning library, scikit-learn [48] (Version 0.17.1). To the best of our knowledge, no detailed comparison study has yet been performed on activity recognition that covers the different categories of classifier algorithms available in scikit-learn.

The same data are input to different classifiers, and the results are stored for analysis. When selecting the classifiers, we used a wide set that includes classical [49] and state-of-the-art [50] techniques. We tested all of the classifiers with various parameters. The classifiers with lower performances were discarded. The following classifiers were finally selected to be studied in more detail.

#### 2.5.1. Distance-Based Methods

These algorithms assume that the data have some type of similarity and relations based on geometric properties and can be grouped according to these patterns.

- The k-nearest neighbors algorithm (k-NN) is one of the simplest and most effective nonparametric machine learning algorithms for classification and regression [51]. The k-NN classifier obtained better results for online recognition when compared to C4.5 decision tree. [52]. After testing several parameters, we eventually used the k-NN algorithm with its default scikit-learn parameters (i.e., k=5 nearest neighbors, uniform weights and Minkowski distance with p=2, which is equivalent to the standard Euclidean metric). The Euclidean distance is the most commonly-used distance metric for continuous variables; however, when working with high-dimensional data (e.g., more than 10 dimensions), it is highly recommended that dimension reduction by means of preprocessing techniques, such as principal component analysis (PCA) or linear discriminant analysis (LDA), be performed. However, for this work, as discussed earlier, we are not testing different feature selection methods or dimension reduction by means of decomposition algorithms for any of the proposed techniques.

#### 2.5.2. Statistical Methods

These techniques assume that data follow a probabilistic function that needs to be inferred.

- When using the Gaussian naive Bayes (Gaussian NB) to work with continuous data, the usual assumption is that the continuous values related to each class are distributed according to a Gaussian distribution [53]. We decided to include Gaussian NB both because it is a well-known technique in machine learning and because it was the best performer without signal segmentation, obtaining an average accuracy of 0.715. However, with the signal segmentation explained in Section 2.3, some of the variables are highly correlated within a class. This negatively affects Gaussian NB, which relies on a model that assumes zero off-diagonal covariance (i.e., no correlations between variables within a class). This technique implements the default version in scikit-learn, where the likelihood of the features is assumed to be Gaussian. In addition to Gaussian NB, we also tested the Bernoulli naive Bayes and multinomial methods. In both cases, these methods achieved worse results compared to Gaussian NB. For exact details on the algorithm used to update feature means and variance online, see the work by [54].
- The LDA technique is a generalization of Fisher’s linear discriminant [55] that is commonly used in statistics, pattern recognition and machine learning to find a linear combination of features that discriminate between two or more classes [56]. LDA can be used as a feature selection algorithm for dimensionality reduction or as a classifier. It is easily computed, has few parameters to tune, is inherently multiclass and has proven to perform very effectively in practice. LDA is closely related to naive Bayes in the sense that both classifiers assume within-class Gaussian distributions. However, LDA relies on a more flexible model that works better with correlations within a class. Therefore, one would expect the LDA results to be better than Gaussian NB under these settings. We also tested the quadratic discriminant analysis, but obtained poor results compared to LDA. We used the default scikit-learn parameters for the comparisons.

#### 2.5.3. Kernel Methods

These models perform pattern analysis based on a kernel function, which is a similarity function over pairs of data points.

- Stochastic gradient descent (SGD) is a simple, but efficient approach to discriminative learning of linear classifiers under convex loss functions, such as logistic regression or support vector machines (linear). Although this technique has long been available in the machine learning community, it has recently received more attention due to its performance with large-scale problems [57]. SGD’s main drawback is that it requires a number of parameters that must be tuned for the method to perform well. For this work, after testing some parameter combinations, we finally used the scikit-learn implementation, which is based on a robust implementation of the averaged stochastic gradient descent algorithm [58]. For this mode, we used the default parameters, setting “hinge” as the loss function, which means that the SGD fits a linear support vector machine and “l2” as the penalty, which is the standard regularization for linear support vector machine models (squared Euclidean norm).
- Support vector machines (SVC linear, SVC RBF) provide state-of-the-art performance for classification, regression and outlier detection, scaling well even with a large-dimensional feature vector [59,60]. The choice of the parameter *C* is critical to achieve a properly trained support vector machine. Our experiments on this problem concluded the best settings are C=0.025 and C=1 for SVC linear and SVC RBF, respectively. The SVC was implemented using libsvm and tested with various kernel functions (i.e., linear, polynomial, radial basis function (RBF) and sigmoid). Finally, based on the test results, the linear and RBF kernel functions were selected for comparison purposes.

#### 2.5.4. Decision Tree-Based Methods

These techniques build a model that predicts a target variable by learning interpretable decision rules inferred from the training data [61]. Using a decision tree as a predictive model is widely used as a decision support tool because they are easily interpreted (i.e., they provide a sequence of decisions to obtain the final classification result).

- Among the different decision tree implementations, we selected one that is widely used: an optimized version of the classification and regression trees (CARTs) algorithm using Gini impurity as a node splitting measure. CART is very similar to C4.5, but differs in its support for numerical target variables. Moreover, it does not compute rule sets.
  We conducted several experiments with decision trees, but the results were not competitive when compared to other techniques, such as random forest or extreme trees. The results obtained with ensembles clearly point out that when executing decision trees, selecting only the relevant features would improve the results significantly. However, because the goal of this work is to compare the performance of algorithms out of the box, we decided not to execute decision trees with a prior feature selection step.

#### 2.5.5. Ensemble Learners

These models combine the predictions of several base estimators built with a given learning algorithm to improve the results and robustness over a single estimator. In [11], the authors evaluated a range of base- and meta-level classifiers and found that combining classifiers using plurality voting provided the best accuracy. When working with these meta-algorithms, it is possible to test a large number of combinations of techniques and parameters. In our work, we ran boosting, bagging and voting with various estimator combinations (k-NN, logistic, random forest and Gaussian); however, we obtained worse results compared to the extra randomized tree ensembles.

- The random forest method consists of an ensemble of decision trees [62]. It combines the predictions made by multiple decision trees, each one generated using a different randomly selected subset of the features [63]. Because random forests are made of several weighted decision trees, they are not easy to interpret (sometimes decisions can be made through voting on contradicting rules). However, they do not require any domain knowledge or complicated parameter settings and perform very well with high-dimensional data. The main parameters to adjust when using these methods are *n_estimators* (i.e., the number of trees in the forest) and *max_features* (the size of the random subsets of features to consider when splitting a node). After testing some values, we set the number of trees to 30 with a maximum depth of 10. Empirically, a good default value is to use $max\_features=\sqrt{n\_features}$ for classification tasks (where *n_features* is the number of features in the data, leading to a *max_features* value for this work of $\sqrt{280}∼17$).
- The extra randomized trees (extra trees) method is similar to the random forest method, but the randomness goes one step further in the way splits are computed [64]. The two main differences are that the extra trees method splits nodes by choosing fully random cut points and grows the trees using the entire learning sample. Similar to the random forest method, the main parameters to adjust are *n_estimators* and *max_features*. In this work, we set the number of trees to 30 and expanded the nodes of the tree until either all pf the leaves were pure or until all of the leaves contained less than 2 samples. As with random forest, *max_features* is set to 17.
- Adaptive boosting (AdaBoost) is a machine-learning meta-algorithm that can be used in conjunction with many other types of learning algorithms to improve their performance [65]. The methods for voting classification algorithms such as bagging and AdaBoost have been very successful for improving results with many different classifiers on both artificial and real-world datasets [66]. Particularly, AdaBoost has been called the best out-of-the-box classifier in the world [67]. The AdaBoost algorithm used is AdaBoost-SAMME.R, a variant of SAMME (Stagewise Additive Modeling using a Multi-class Exponential loss function) which is recommended for continuous multi-class domains [68]. We used AdaBoost to test the performance of random forest (AdaBoost random forest) and extra randomized trees (AdaBoost extra trees) algorithms. The parameters for the tree-based ensembles were the same as described earlier.

#### 2.5.6. Deep Learning

These techniques are an extension or a re-branding of neural networks [69]. They are based on the idea of modeling problems with high-level abstractions in data by using multiple processing layers that basically perform multiple non-linear transformations. These multiple transformations are organized hierarchically, with each layer processing the outputs of the previous layer, exploiting the idea of incremental learning, in which more abstract concepts are learned from the lower level ones. The processing layers can have various architectures, such as deep neural networks, convolutional deep neural networks, deep belief networks or recurrent neural networks. The idea of using deep learning layers is very interesting to researchers in many fields because these techniques can automatically build high-level representations of the raw input, offering a more generic solution because the feature construction process can be fully automated. Deep learning techniques have been successfully applied to many different fields, including computer vision, natural language processing, speech recognition, and so on, and have achieved state-of-the-art results on various tasks [70,71,72]. Regarding activity recognition, deep learning techniques have been applied in a few works; a representative set can be found in [26,27,28,29,30,31].

**Figure 4:**
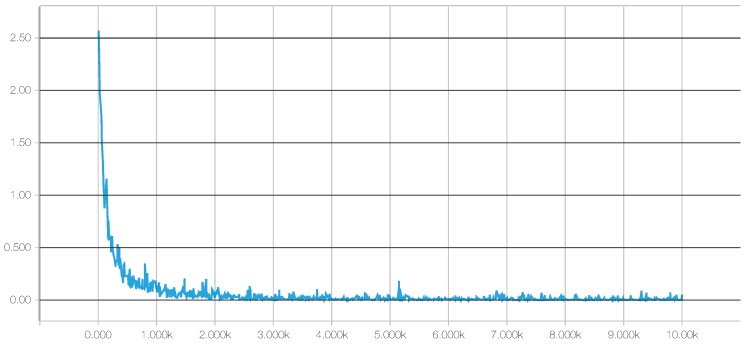
Evolution of the logistic regression loss function along iterations of the deep neural network training process, generated with TensorBoard.

**Figure 5:**
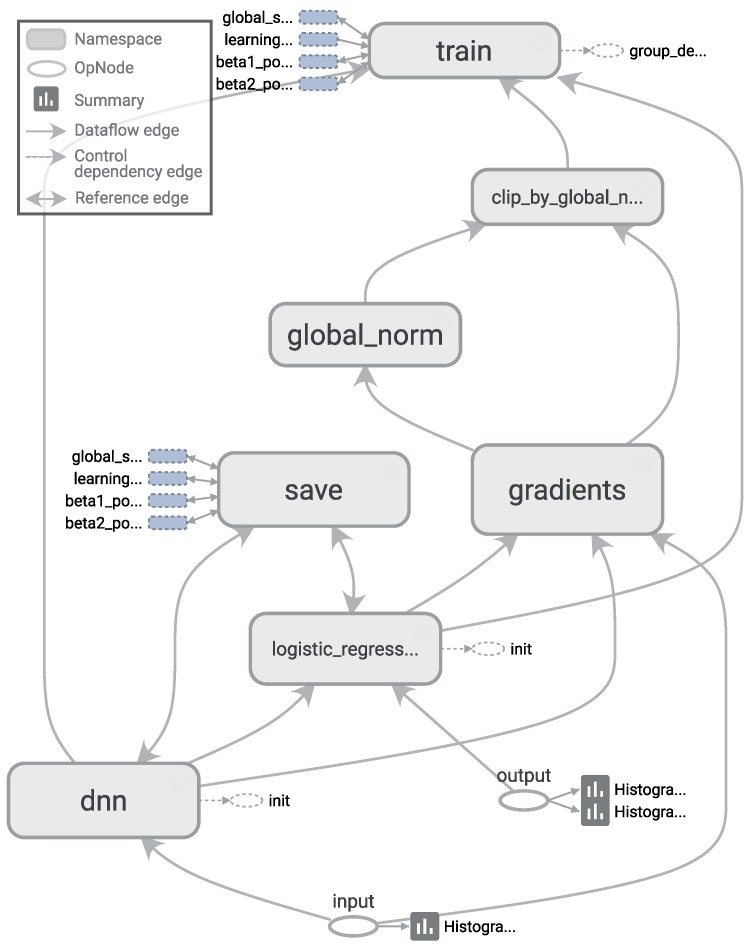
Architecture of a TensorFlow graph for training a deep neural network, generated with TensorBoard.

- For a deep neural network (DNN), we chose the TensorFlow framework, recently introduced by Google (Mountain View, CA, USA) [73]. Additionally, to achieve a better integration with scikit-learn, we used the skflow package, which provides a simplified interface for accessing and mimicking TensorFlow scikit. As mentioned before, deep learning techniques have the advantage of being able to build high-level representations of the raw input data without the need for data processing and segmentation. While this is an interesting approach, we have identified a very high impact of the network architecture on the results, a behavior consistent with what we have found in the literature [32], including the fact that some architectures do not work at all. Therefore, in this work, to create an objective comparison, we used the same preprocessed data to test all of the classifiers, including the DNNs. The main motivation for testing these techniques with preprocessed data is to investigate whether their ability to extract and/or transform features among the 280 attributes translates into competitive classification performances. Given the importance of the architecture design, for this work, we have tested numerous topologies (combinations of tensors and dense and convolutional layers). However, after running a large set of experiments, we observed through the evolution of the logistic regression loss function that simple architectures were sufficient to converge within a few iterations obtaining competitive results, as shown in Figure 4. Adding more complexity to the topology required considerably more computation time, all to achieve only very small improvements in accuracy (or even no improvement at all due to overfitting). The results of our tests with different architectures pointed out two things: (1) incorrect topology designs dramatically affect the results; and (2) the results obtained with simple architectures were probably so close to the global optimum that adding more complexity in the layers did not lead to any significant improvement.
  Consequently, for comparison purposes, we finally selected two configurations: DNN 1L, which has 1 hidden dense layer with 280 nodes, and DNN 2L, which has 2 hidden dense layers with 560 and 280 nodes, respectively. For both configurations, we used a logistic regression as the cost function, 10,000 iterations with the Adam optimizer, a dropout probability of 0.5 and a learning rate of 0.001. The computational graph for training such a deep neural network, as generated by TensorBoard, is shown in Figure 5.

### 2.6. Parameter Setup

Table 4 shows the parameters used when running each of the classifiers. These parameter values were determined after a previous phase of manual exploration. The table also indicates whether the input data were normalized to the range [0, 1] after training the classifier. Note that while both normalized and non-normalized data were tested with all algorithms, only those configurations that led to higher accuracies are detailed in this paper. Although they are not shown in the table, random seeds are chosen randomly; thus, they are different for each run. Parameters that are not shown in the table were set to their default values.

### 2.7. Quality Metrics

For each individual run of all of the experiments conducted, we collected accuracy, precision, recall and $F_{\beta }$ scores, with β={1,2}. The accuracy metric is simply the percentage of correctly-classified instances. Although the dataset used for this work is not perfectly balanced, because we are working with 12 classes, we consider that average accuracy metric to be both a simple and appropriate metric for representing the classifiers’ overall performances. For this reason, and to aid readability, we summarized only the accuracy metric plus variance for the preliminary evaluation. Of course, we also analyzed the confusion matrix, precision, recall, $F_{1}$ and $F_{2}$, but without finding any significant results to discuss.

For the selected classifiers, we have included more detail about the confusion matrix, precision (Equation (2)), recall (Equation (3)) and $F_{\beta }$ (Equation (4)) scores, with β=1 and β=2. Because this is a multiclass problem, these metrics are obtained by weighted averaging. The following equations describe how each of the previous metrics is computed:

(2) $$P\left(y,\hat{y}\right)=k\sum_{l\in L}\left|{\hat{y}}_{l}\right|\frac{|y_{l}\cap {\hat{y}}_{l}|}{|y_{l}|}$$

(3) $$R\left(y,\hat{y}\right)=k\sum_{l\in L}\left|{\hat{y}}_{l}\right|\frac{|y_{l}\cap {\hat{y}}_{l}|}{|{\hat{y}}_{l}|}$$

(4) $$F_{\beta }\left(y,\hat{y}\right)=k\sum_{l\in L}\left|{\hat{y}}_{l}\right|\left(1+\beta^{2}\right)\frac{P\left(y_{l},{\hat{y}}_{l}\right)\times R\left(y_{l},{\hat{y}}_{l}\right)}{\beta^{2}P\left(y_{l},{\hat{y}}_{l}\right)+R\left(y_{l},{\hat{y}}_{l}\right)}$$

These equations conform to the following conventions:

- *y* is the set of predicted (sample, label) pairs.
- $\hat{y}$ is the set of true (sample, label) pairs.
- *L* is the set of labels.
- $y_{l}$ and ${\hat{y}}_{l}$ are, respectively, the subsets of *y* and $\hat{y}$ with label *l*.
- $k=(\sum_{l\in L}|{\hat{y}}_{l}{\left|\right)}^{-1}$

Another commonly-used quality metric is the receiver operating characteristic (ROC) metric; however, for this work, we did not analyze the ROC curves, because this is a multiclass problem. First, ROC curves, which generally work well for visualization, would be difficult to visualize for 12 classes, requiring the volume under the ROC surfaces metric (VUS) or Cobweb diagrams. Second, for each class, a pairwise comparison of the performance of that class versus all of the other classes would be required [74,75].

## 3. Results

This section describes the results obtained from the evaluation of the different classifiers.

### 3.1. Preliminary Evaluation

The purpose of the preliminary evaluation is to quickly evaluate the performance of the very different techniques included. To do this, we used small percentages of the training data, thus decreasing the required training time. In particular, we first tried running the experiments with 5% of the training data. Additionally, to check whether the subset size affects the accuracy, we also compared the performance of the various techniques using sampled subsets of 10%, 1%, 0.5%, 0.1%, 0.05%, 0.01%, 0.005% and 0.001% of the training set, respectively.

The reduced training subset is built by random sampling without replacement. Given that the training subset is random while most algorithms are stochastic, the performance for each technique and subject is computed by taking the average results from 50 runs with different random seeds (two random seeds are used, one for sampling the training set and the other for the classifier initialization).

### 3.2. Classifiers’ Performances

The average performances for each classifier were studied by computing the accuracy on a per-classifier basis. During the experiments, a different random 5% of the training data were sampled for each run, and 50 runs were executed for each subject, totaling 400 runs for each classifier. Because we used LOSO cross-validation, the training set for each subject contains only data from other subjects.

**Figure 6:**
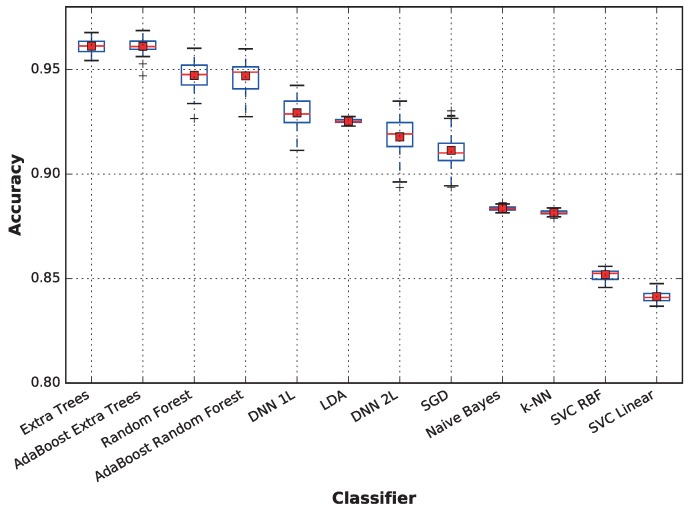
The distribution of accuracies along 50 runs for each classifier. The data represent the average accuracies for all subjects. The results were obtained by training each model 50 times per subject with 5% of the training data, using LOSO cross-validation.

Figure 6 displays the aggregated accuracy performance of each classifier. A more detailed analysis of the average accuracy, as well as the standard deviation are provided on a per-subject basis in Table 5, where the best classifier for each subject is highlighted in bold text. The figure shows that the extra trees technique is the best for Subjects 6 and 8; LDA provides the highest accuracies for Subjects 3, 4 and 7; and the deep neural network is the best choice for Subjects 1 and 2. Surprisingly, while SVC with the RBF kernel provides low accuracy on average, it outperforms the other classifiers when predicting the activity for Subject 5.

When considering the average performance, techniques based on ensembles of decision trees outperform the other techniques, followed by deep neural networks and linear discriminant analysis, which also has a very low variance. On the other hand, support vector machines obtain a very low performance; both SVC with linear and RBF kernels and k-NN and naive Bayes result in below-average accuracy when there are no competitive results for any subject.

The results of the meta-classifiers (AdaBoost over both extra trees and random forest) show that they do not significantly affect performance. For the remainder of this paper, the following classifiers will be omitted from the comparisons: AdaBoost extra trees and AdaBoost random forest, because they provide results very similar to those not using meta classifiers; random forest, because it is dominated by extra trees, another decision-tree based ensemble; DNN 2L (featuring two dense layers), because it provides no advantage over DNN 1L and achieves slightly worse results; and naive Bayes, k-NN and SVC, because they do not achieve competitive performances compared to the other classifiers, obtaining significantly lower averages and medians. Nevertheless, in the paper conclusions, we will place all classifiers in a concept map showing how they compare relative to each other both in accuracy and in sensitivity to the dataset size.

**Figure 7:**
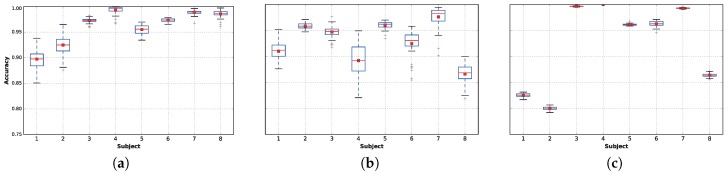
Distribution of accuracy over the test set for each subject, for the outperforming classifiers, using 5% of the training data. Each distribution results from the execution of 50 runs. (**a**) Extra trees; (**b**) DNN 1L; (**c**) LDA.

For the remaining classifiers, extra trees, DNN 1L (featuring one dense layer) and LDA, Figure 7 plots the accuracy distributions for each subject when using only 5% of the data. In general, extra trees (Figure 7a) provides very high accuracies for most of the subjects, although DNN (Figure 7b) provides better results for Subjects 1 and 2. Finally, LDA (Figure 7c) provides extremely high accuracies for Subjects 3 and 7 and perfect accuracy in all 50 runs for Subject 4. The best results found in this work are slightly better than the ones presented in Table 4.9 of [18]; however, in comparing the results, it is important to note that [18] included Subject 9, while we decided to remove him from this work because he performed only the rope jumping activity for 64.90 s, as reflected in Table 3. Nevertheless, because Subject 9’s task is quite easy to classify (96% average accuracy), if we had included him in the training sets, our overall results would have improved, outperforming the results presented in [18] even more.

### 3.3. Sensitivity to Training Sample Size

In this paper, we also investigated how different classifiers perform when the training dataset is significantly small. This is an interesting research topic for three reasons. First, working with a small sample of the datasets means less power consumption when processing and transmitting data, and a low energy consumption is vital for most wearable devices. Second, it allows us to simulate a cold start situation in which only a few users have been used for training, but new users are starting to use the system. Third, when working with large datasets, reducing the amount of data required would enable the processing of big data, effectively reducing its size by several orders of magnitude simply by performing random sampling of some tranches. We hypothesize that the performance of classifiers is not equal (given the correlations between different training sample sizes and accuracy), but we wanted to validate this hypothesis and explore these effects for the different chosen classifiers.

**Figure 8:**
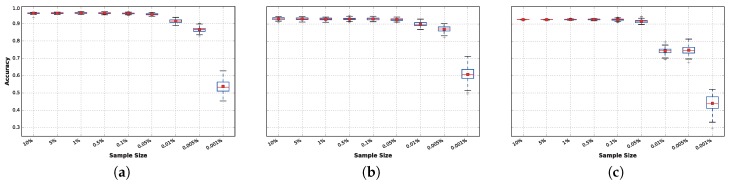
How training sample size affects the accuracy distributions, aggregated for all subjects, for the high-performing classifiers. (**a**) Extra trees; (**b**) DNN 1L; (**c**) LDA.

In Figure 8, it can be observed that after 0.05%, the accuracy decreases if the sample size is reduced further, resulting in statistically-significant differences in performance. The results obtained within the range of 10% to 0.05% have very small variations that can be explained by the random seed used for each run. The reason these results are nearly identical is because the FFT collects data from overlapping sliding windows of 5.12 s. Therefore, even when instances are removed from the training set, there is no real loss of data. Loss of data truly begins to affect the tested classifiers in experiments where less than 0.05% of the data is available.

However, this effect varies from one classifier to another. In general, there is no significant difference in accuracy for sample sizes as low as 0.05% of the complete training dataset for any of the techniques. However, this behavior changes when the training sample size is reduced to 0.01%: At this point, DNN (Figure 8b) and extra trees (Figure 8a) suffer from a small reduction in accuracy, while this reduction is significantly magnified in the case of LDA (Figure 8c), for which the average accuracy drops to approximately 75%.

When reducing the data further (from 0.01% to 0.001%), significant performance differences arise. These are particularly noticeable for LDA, while DNN is the least affected technique. It is noteworthy that the best classifiers obtained results above 90% accuracy on average, even with only 0.005% of the continuous data available, which corresponds to collecting only approximately 9.17 consecutive seconds of each activity per subject.

### 3.4. Analysis of the Results

**Figure 9:**
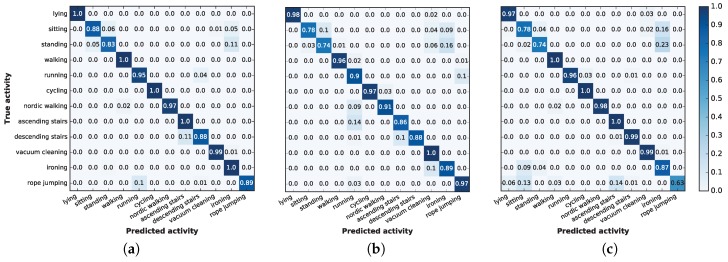
Confusion matrices for the 12 activities aggregated for all subjects, for the high-performing classifiers. (**a**) Extra trees; (**b**) DNN 1L; (**c**) LDA.

A more exhaustive summary of the results is shown in Figure 9, which depicts the confusion matrices for the three selected machine learning techniques: extra trees, deep neural networks and linear discriminant analysis. The confusion matrices are obtained from a random execution using 5% of the training data, and the values shown are obtained by aggregating the results for the eight subjects and, later, normalizing the values for each class.

The high accuracy levels are reflected in the three matrices by the very high values in the main diagonal. Many of these values are one, implying that certain activities were perfectly recognized.

In general, a study of the confusion matrices shows that, in all cases, standing is sometimes classified as ironing; however, this misclassification never occurs the other way around. This particular misclassification is particularly common using LDA, which incorrectly recognized 23% of the instances. However, this confusion is reasonable: the protocol defined that people performing the standing activity could still gesticulate, and some hand or arm movements may resemble the act of ironing.

Extra trees and DNN sometimes misclassify the descending stairs action as an ascending stairs action. Moreover, DNN incorrectly classifies ascending stairs or Nordic walking as running, while extra trees confuses these activities with rope jumping. However, these issues occur only 10% to 15% of the time, and again, the activities are remarkably similar; therefore, we consider that these misclassifications are reasonable.

Finally, using LDA led to some remarkable mistakes that cannot be explained by the nature of the activities. In particular, it classified both ironing and jumping rope as sitting, even though these activities are significantly different. The first confusion is symmetric: 16% of the time when a subject was sitting, LDA recognized the activity as ironing. However, LDA confuses jumping rope with several activities, some of which are quite dissimilar. The reason LDA confuses jumping rope with other activities is because only five out eight subjects performed this activity, and those who did spent less time (on average) compared to the time they spent on other activities (one jumped rope for only 2.55 s). This finding is in accordance with our previous findings: LDA requires more data than the other selected methods to achieve an accurate prediction.

For each of these classifiers, other performance metrics, including precision, recall, $F_{1}$ score and $F_{2}$ score, are shown in Table 6. These metrics were obtained using both 5% and 0.001% of the training data and are the result of 50 runs per subject. The table shows the mean, as well as the standard deviation (enclosed within parentheses).

For all metrics, when 5% of the training data are used, extra trees outperforms DNN, which outperforms LDA. Still, the variance is smaller for both the latter methods, meaning that their performance is more stable along the 50 runs. However, when the sample size is limited to 0.01% of the training data, DNN begins to outperform extra trees, an effect that can be observed in all of the metrics.

## 4. Conclusions and Future Work

In this work, we compared different classification techniques for automatic cross-person activity recognition under different scenarios where more or less information is available. We used data from the PAMAP2 dataset collected from eight participants performing 12 activities with wearable devices as explained in detail in Section 2. First, we performed some preprocessing to remove unneeded data and to insert missing values. Then, we extracted time and frequency features from a 5.12-s sliding window using a fast Fourier transform (FFT). As a result, each physiological feature in the preprocessed dataset was replaced with seven features in the resulting dataset (average, median, variance, maximum, minimum, 25th percentile and 75th percentile).

After obtaining the transformed dataset, we compared how the best classifier techniques performed under scenarios with different amounts of training data. We tested sampling rates of 10%, 1%, 0.5%, 0.1%, 0.05%, 0.01%, 0.005% and 0.001% of the complete training set. For each scenario, we tested k-nearest neighbors, Gaussian naive Bayes, linear discriminant analysis, stochastic gradient descent, support vector machines with several kernel functions, decision trees, random forest, extra randomized trees, adaptive boosting and deep neural networks (TensorFlow), among other algorithms. For replication purposes, all of these algorithms were implemented using the scikit-learn library as described in detail in Section 2.5.

When the goal is cross-person prediction, subject-specific training cannot be performed; otherwise, there would be no unseen data for new users. For this reason, we used the leave-one-subject-out (LOSO) cross-validation technique. This evaluation methodology splits the dataset into *N* folds (here, N=8 subjects), using N−1 folds as the training set and the remaining fold as the test set. Using this methodology, we can guarantee that the classifier learns nothing about the remaining subject being evaluated for prediction. Consequently, the possible learning biases decrease, and we can obtain reliable results when performing cross-person prediction.

After conducting a preliminary set of experiments, we concluded that with our experimental setup, extra randomized trees performed extremely well, outperforming state-of-the-art results described in previous works [18]. Other related work using the same dataset and performing feature selection with random forest have reported an overall accuracy of 97.9% [76]. However, because their results are not evaluated using LOSO, their training set includes subject information, and therefore, their results are not directly comparable to ours. The results obtained with extra randomized trees were so good (96.1%±0.009% on average) that using AdaBoost did not improve them. The results of the other classifiers are in accordance with reports by previous works [18,45,77]. For instance, when analyzing the confusion matrix, incorrect predictions only appear in neighbor (similar) classes. Random forest performed quite well, achieving second place in overall average accuracy, which is unsurprising considering that extra randomized trees are based on random forest. Again, this finding confirms other results from the literature [18,24], where the authors stated that, overall, boosted decision tree classifiers and k-NN attained the best performances.

Analyzing the results in further detail, we found that LDA was able to achieve the best results in three of eight subjects (those whose activities were easiest to predict), and LDA obtained an outstanding 100% for Subject 4. The deep neural networks were able to achieve the best results for Subjects 1 and 2, whose activities are more diverse and difficult to predict. At this point, we can confirm that we found competitive results for deep neural networks and have successfully used TensorFlow to classify physical activities. To our knowledge, this is one of the first works in the literature to do so.

However, as mentioned above, more complex deep architectures did not translate to better results, mainly because, after processing the dataset, the problem was not complex enough and because deeper architectures tend to overfit the training sets. We note that these simple architectures are conceptually not pure deep learning. Nevertheless, the results attained in this work outperform previous works using deep learning over raw data [32], and it is even more interesting considering that our results are cross-subject. We think that this topic itself is significantly interesting and extensive, and we have decided to leave a whole analysis of the behavior of deep learning techniques with raw/preprocessed data as a future work.

After conducting a sensitivity analysis of different training sample sizes, we concluded that deep neural networks were the techniques whose average accuracies were least affected (see Table 6). Another interesting conclusion regarding training sample size is that sample sizes of 0.05% obtain results very similar to experiments using sample sizes of 10%. This occurs for two reasons: there is intrinsic redundancy in the data, and this type of data is easily generalizable from a small sample. The most important consequence of this finding is that, using this dataset, we could obtain essentially identical results by collecting only 22.67% of continuous data from the devices rather than the complete set of labeled activities. It is remarkable that the best classifiers obtained results above 90% accuracy on average using only 4.53% of the complete dataset, which corresponds to an average of only 9.17 consecutive seconds of each activity by each subject.

**Figure 10:**
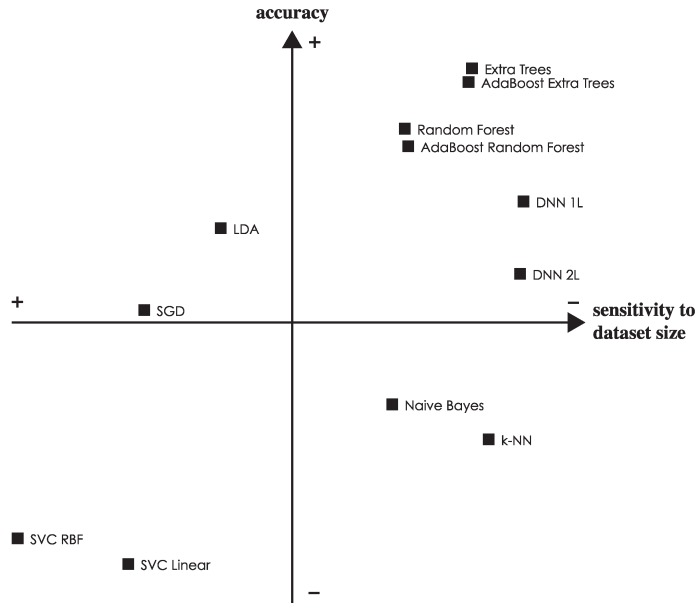
Concept map showing the relative performance of each classifier both in terms of accuracy and sensitivity to the dataset size. In the former metric, more (up) is better, while in the latter, less (right) is better, as lower values represent less variance in the accuracy when reducing the dataset size.

To put it together, Figure 10 shows the relative strength of each classifier both in terms of accuracy and sensitivity to the dataset size (i.e., variance in the accuracy as the dataset is reduced). The best performing classifiers are shown in the top right quadrant, and we can see how extra trees and DNN 1L are non-dominated choices.

As said before, this is one of the first works using the scikit-learn library for physical activity recognition. After working extensively with scikit-learn, we can strongly recommend it to the scientific community. The library reduces the overall development time and simplifies the experimental replication process. Moreover, because the programming language is Python, many additional libraries are available, which are, based on our experience, much more powerful tools than WEKA.

As future work, in addition to further investigating deep learning and raw data, as mentioned above, there are two other correlated research lines that require attention. First, there is a need to test more datasets collected from smartphones or wearable devices, such as [17,47,78,79] or collected by our team. Second, there is a need to build a benchmark tool that includes more classifiers and feature selection methods. We also would like to compare performances between datasets of different physical activities using template matching, as observed in [47]. After accomplishing these objectives, we would like to release this benchmark as open-source code, including all of the implemented feature selection methods and classifiers and the collected datasets.

We are aware that the levels of accuracy found in this work may decrease in the real world; here, sensors position might vary or interference from other devices may occur. In future work, it would be ideal to compare laboratory results with subjects in real-world environments, as studied in [80].

Regarding feature selection, issues such as finding the optimal combination of the time and frequency domain for features and analyzing how feature selection affects each classifier’s performance should be explored. In this sense, in addition to the features used in this paper, we could test the signal-magnitude area [81], energy [11], entropy [82], binned distribution histogram of FFT and time between peaks [83], among others.

Finally, as our main conclusion, when performing cross-subject physical activity recognition in situations with limited data, it is possible to obtain high prediction accuracies (>90%) using the extra randomized trees, random forest and deep neural networks method. Using basic parameters, these methods perform well when generalizing among subjects and with limited data. Other techniques, such as linear discriminant analysis, Gaussian naive Bayes or k-nearest neighbors, support vector machines (with linear and radial kernel functions) or stochastic gradient descent, are not able to achieve high prediction accuracies with such limited data. It is also important to mention that normalizing the input data may positively or negatively affect classifiers. Normalizing the input data is a required step for some classifiers. At present, we can confirm these findings only under the conditions explained in Section 2, but in the future, we will attempt to extrapolate them to different physical activity datasets.

## Figures and Tables

**Table 1:** Physiological information about the subjects participating in the data acquisition protocol. HR, heart rate.

#	Sex	Age (years)	Height (cm)	Weight (kg)	Resting HR (bpm)	Max HR (bpm)	Dominant Hand
1	Male	27	182	83	75	193	right
2	Female	25	169	78	74	195	right
3	Male	31	187	92	68	189	right
4	Male	24	194	95	58	196	right
5	Male	26	180	73	70	194	right
6	Male	26	183	69	60	194	right
7	Male	23	173	86	60	197	right
8	Male	32	179	87	66	188	left
9	Male	31	168	65	54	189	right

**Table 2:** Protocol for data collection, including the activity, the estimated effort (through the metabolic equivalent) and the time each activity must be performed. MET, metabolic equivalent.

#	Activity	Code	Effort (MET)	Time (min)
1	lying	07011	1.0	3
2	sitting	09040	1.8	3
3	standing	09050	1.8	3
4	ironing	05070	2.3	3
5	break			1
6	vacuuming	05043	3.5	3
7	break			1
8	ascending stairs	17130	8.0	3
9	break			2
10	descending stairs	17070	3.0	3
11	break			1
12	ascending stairs	17130	8.0	3
13	descending stairs	17070	3.0	3
14	break			2
15	walking	17190, 17200	3.3–3.8	3
16	break			1
17	Nordic walking	?	5.0–6.0	3
18	break			1
19	cycling	01010	4.0	3
20	break			1
21	running	12020, 12030	7.0–8.0	3
22	break			2
23	jumping rope	15551, 15552	8.0–10.0	3

**Table 3:** Actual time (in seconds) spent by the subjects in each activity (this may differ from the protocol). Sub., subject.

	Sub. 1	Sub. 2	Sub. 3	Sub. 4	Sub. 5	Sub. 6	Sub. 7	Sub. 8	Sub. 9
Lying	271.86	234.29	220.43	230.46	236.98	233.39	256.10	241.64	0.00
Sitting	234.79	223.44	287.60	254.91	268.63	230.40	122.81	229.22	0.00
Standing	217.16	255.75	205.32	247.05	221.31	243.55	257.50	251.59	0.00
Walking	222.52	325.32	290.35	319.31	320.32	257.20	337.19	315.32	0.00
Running	212.64	92.37	0.00	0.00	246.45	228.24	36.91	165.31	0.00
cycling	235.74	251.07	0.00	226.98	245.76	204.85	226.79	254.74	0.00
Nordic walk	202.64	297.38	0.00	275.32	262.70	266.85	287.24	288.87	0.00
Ascending stairs	158.88	173.40	103.87	166.92	142.79	132.89	176.44	116.81	0.00
Descending stairs	148.97	152.11	152.72	142.83	127.25	112.70	116.16	96.53	0.00
Vacuuming	229.40	206.82	203.24	200.36	244.44	210.77	215.51	242.91	0.00
Ironing	235.72	288.79	279.74	249.94	330.33	377.43	294.98	329.89	0.00
Jumping rope	129.11	132.61	0.00	0.00	77.32	2.55	0.00	88.05	64.90

**Table 4:** Classifiers used in scikit-learn along with the values set for their parameters and whether data were previously normalized in the range [0,1].

Classifier	Normalized
**sklearn.neighbors.KNeighborsClassifier** (k = 5)	Yes
**sklearn.naive_bayes.GaussianNB**	No
**sklearn.discriminant_analysis.LinearDiscriminantAnalysis**	No
**sklearn.linear_model.SGDClassifier** (loss = hinge, penalty = 12)	Yes
**sklearn.svm.SVC** (kernel = linear, max_iter = − 1, C = 0.025)	Yes
**sklearn.svm.SVC** (kernel = rbf, max_iter = −1, C = 1)	Yes
**sklearn.ensemble.ExtraTreesClassifier** (n_estimators = 30)	No
**sklearn.ensemble.RandomForestClassifier** (n_estimators = 30, max_depth = 10)	No
**sklearn.ensemble.AdaBoostClassifier** (ExtraTreesClassifier, n_estimators = 30)	No
**sklearn.ensemble.AdaBoostClassifier** (RandomForestClassifier, n_estimators = 30, max_depth = 10)	No
**skflow.ops.dnn** ([280], logistic_regression, keep_prob = 0.5, steps = 10000, optimizer = Adam, learning_rate = 0.001)	Yes
**skflow.ops.dnn** ([560, 280], logistic_regression, keep_prob = 0.5, steps = 10000, optimizer = Adam, learning_rate = 0.001)	Yes

**Table 5:** Average accuracy (and standard deviation) for each classifier and subject when using 5% of the training data. The bold text indicates the classifiers that outperformed others for each subject. ET, extra trees; RF, random forest; SGD, stochastic gradient descent.

Classifier	Accuracy								Average
	Sub. 1	Sub. 2	Sub. 3	Sub. 4	Sub. 5	Sub. 6	Sub. 7	Sub. 8	
Extra Trees	0.897 (0.019)	0.925 (0.018)	0.973 (0.004)	0.993 (0.008)	0.955 (0.009)	**0.973** (0.003)	0.989 (0.004)	**0.986** (0.007)	0.961 (0.009)
AdaBoost (ET)	0.900 (0.018)	0.922 (0.022)	0.972 (0.004)	0.993 (0.009)	0.954 (0.008)	**0.973** (0.003)	0.989 (0.004)	0.985 (0.008)	0.961 (0.009)
Random Forest	0.874 (0.012)	0.895 (0.045)	0.955 (0.010)	0.982 (0.020)	0.953 (0.014)	0.966 (0.008)	0.975 (0.012)	0.978 (0.005)	0.947 (0.016)
AdaBoost (RF)	0.873 (0.011)	0.897 (0.042)	0.955 (0.010)	0.983 (0.021)	0.951 (0.017)	0.966 (0.007)	0.975 (0.012)	0.977 (0.005)	0.947 (0.015)
DNN 1L	**0.910** (0.016)	**0.959** (0.006)	0.947 (0.012)	0.892 (0.033)	0.960 (0.007)	0.925 (0.024)	0.976 (0.002)	0.866 (0.019)	0.929 (0.017)
LDA	0.825 (0.003)	0.800 (0.003)	**0.997** (0.001)	**1.000** (0.000)	0.961 (0.002)	0.963 (0.005)	**0.993** (0.001)	0.864 (0.003)	0.925 (0.002)
DNN 2L	0.908 (0.021)	0.956 (0.008)	0.913 (0.027)	0.870 (0.053)	0.948 (0.012)	0.934 (0.025)	0.955 (0.031)	0.858 (0.029)	0.918 (0.026)
SGD	0.862 (0.011)	0.826 (0.043)	0.973 (0.006)	0.923 (0.025)	0.971 (0.005)	0.922 (0.014)	0.983 (0.001)	0.831 (0.034)	0.911 (0.017)
Naive Bayes	0.821 (0.006)	0.604 (0.004)	0.961 (0.001)	0.991 (0.001)	0.922 (0.002)	0.955 (0.001)	0.941 (0.002)	0.874 (0.003)	0.884 (0.003)
k-NN	0.860 (0.003)	0.950 (0.001)	0.855 (0.004)	0.719 (0.005)	0.942 (0.002)	0.886 (0.003)	0.955 (0.003)	0.886 (0.002)	0.882 (0.003)
SVC RBF	0.865 (0.003)	0.864 (0.017)	0.815 (0.006)	0.619 (0.006)	**0.975** (0.001)	0.933 (0.002)	0.869 (0.005)	0.876 (0.005)	0.852 (0.006)
SVC Linear	0.858 (0.004)	0.820 (0.011)	0.816 (0.010)	0.601 (0.009)	0.974 (0.002)	0.917 (0.004)	0.883 (0.007)	0.861 (0.007)	0.841 (0.007)

**Table 6:** Performance metrics mean (and standard deviation) for the high-performing classifiers, comparing sample sizes of 5% and 0.001%. Higher values (better) are displayed in boldface.

		Extra Trees	DNN	LDA
5%	Precision	**0.967** (0.003)	0.949 (0.005)	0.926 (0.001)
	Recall	**0.961** (0.003)	0.929 (0.007)	0.925 (0.001)
	$F_{1}$ Score	**0.959** (0.004)	0.927 (0.007)	0.912 (0.001)
	$F_{2}$ Score	**0.960** (0.004)	0.927 (0.007)	0.917 (0.001)
0.001%	Precision	0.476 (0.005)	**0.547** (0.006)	0.384 (0.005)
	Recall	0.538 (0.004)	**0.607** (0.005)	0.440 (0.005)
	$F_{1}$ Score	0.455 (0.005)	**0.536** (0.006)	0.361 (0.005)
	$F_{2}$ Score	0.491 (0.004)	**0.567** (0.005)	0.393 (0.005)
